# Climate-driven *in situ* trait variation in an annual ruderal grass across Europe

**DOI:** 10.1093/aob/mcag164

**Published:** 2026-06-27

**Authors:** Helene Villhauer, Timo Hellwig, Anna Elisabeth Backhaus, Przemyslaw Baranow, Albane Bignon, José Manuel Blanco Moreno, Denise V M Blume, Anna Bomanowska, Milan Brankov, Luís P da Silva, Andreas D Drouzas, Walter Durka, Severin Einspanier, Arndt Hampe, Miloš Ilić, Konrad Kaczmarek, Abdenour Kheloufi, Marta Kolanowska, Kamil Konowalik, Izabela Krzemińska, Sandy Jan Labarosa, Mathieu Leclerc, Matej Lexa, Lahouaria Mounia Mansouri, Víctor Manzanares-Vázquez, Sabine Metzger, Nadine Mitschunas, Jonathan Moore, Ondřej Mudrák, Monika Myśliwy, Pablo Neira, Nebojša Nikolić, Agnieszka Nobis, Marcin Nobis, Artur Nosalewicz, Sławomir Nowak, Sylvain Pincebourde, Boris Radak, Agnieszka Rewicz, Encarna Rodríguez-García, Carmen Romeralo, Aritz Royo-Esnal, Arne Saatkamp, Ali Sahri, Francesco Santi, Henryk Straube, Jannis Straube, Katalin Szitár, Joel Torra, Markus Wagner, Adrian Wysocki, Anis Edena Zarantonello, Stanislav Kopriva, Maria von Korff, Anna Bucharova

**Affiliations:** Conservation Biology, Department of Biology, Philipps University Marburg, Marburg 35043, Germany; Institute of Plant Genetics, Department of Biology, Heinrich-Heine-University Düsseldorf, Düsseldorf 40225, Germany; Leibniz Institute of Plant Genetics and Crop Plant Research (IPK) OT Gatersleben, Seeland 06466, Germany; International Centre for Agricultural Research in the Dry Areas (ICARDA), Rabat 1000, Morocco; Department of Plant Taxonomy and Nature Conservation, Faculty of Biology, University of Gdańsk, Gdańsk 80-308, Poland; Laboratoire de Biométrie et Biologie Evolutive (LBBE), UMR 5558, CNRS, Université Claude Bernard Lyon 1, Villeurbanne 69100, France; Departament de Biologia Evolutiva, Ecologia i Ciències Ambientals, Facultat de Biologia, Universitat de Barcelona (UB), Barcelona 08028, Spain; Institut de Recerca de la Biodiversitat (IRBio), Universitat de Barcelona (UB), Barcelona 08028, Spain; Faculty of Science, Department of Plant and Environmental Sciences, Section for Plant Biochemistry, University of Copenhagen, Frederiksberg 1871, Denmark; Department of Geobotany and Plant Ecology, Faculty of Biology and Environmental Protection, University of Lodz, Lodz 90-237, Poland; Agroecology and Cropping Practices Group, Maize Research Institute Zemun Polje, Belgrade 11185, Serbia; CIBIO, Centro de Investigação em Biodiversidade e Recursos Genéticos, InBIO Laboratório Associado, Campus de Vairão, Universidade do Porto, Vairão 4485-661, Portugal; BIOPOLIS Program in Genomics, Biodiversity and Land Planning, CIBIO, Campus de Vairão, Vairão 4485-661, Portugal; Laboratory of Systematic Botany and Phytogeography, School of Biology, Aristotle University of Thessaloniki, Thessaloniki GR-54124, Greece; Department of Community Ecology (BZF), Helmholtz Centre for Environmental Research – UFZ, Halle 06120, Germany; German Centre for Integrative Biodiversity Research (iDiv) Halle–Jena–Leipzig, Leipzig 04103, Germany; Department of Phytopathology and Crop Protection, Institute of Phytopathology, Faculty of Agricultural and Nutritional Sciences, Kiel University, Kiel 24098, Germany; INRAE, University of Bordeaux, BIOGECO, Cestas F-33610, France; Department of Biology and Ecology, Faculty of Sciences, University of Novi Sad, Novi Sad 21000, Serbia; Department of Geobotany and Plant Ecology, Faculty of Biology and Environmental Protection, University of Lodz, Lodz 90-237, Poland; Department of Ecology and Environment, University of Batna 2, Batna 05078, Algeria; Department of Geobotany and Plant Ecology, Faculty of Biology and Environmental Protection, University of Lodz, Lodz 90-237, Poland; Department of Botany and Plant Ecology, Wrocław University of Environmental and Life Sciences, Wrocław 50-363, Poland; Institute of Agrophysics, Polish Academy of Sciences, Lublin 20-290, Poland; Institute of Plant Genetics, Department of Biology, Heinrich-Heine-University Düsseldorf, Düsseldorf 40225, Germany; Institut de Recherche sur la Biologie de l'Insecte, UMR 7261, CNRS – Université de Tours, Tours 37200, France; Faculty of Informatics, Masaryk University, Brno 60200, Czech Republic; Department of Ecology and Environment, University of Batna 2, Batna 05078, Algeria; Department of Research and Development, Coccosphere Environmental Analysis, Málaga 29120, Spain; Institute for Plant Sciences, Cluster of Excellence on Plant Sciences (CEPLAS), University of Cologne, Cologne 50674, Germany; UK Centre for Ecology & Hydrology, Benson Lane, Oxfordshire OX10 8BB, UK; Tropic Biosciences Ltd., Norwich Research Park Innovation Centre, Norwich NR4 7GJ, UK; Institute for Environmental Studies, Charles University, Prague 128 01, Czech Republic; Institute of Soil Biology and Biochemistry, Biology Centre of the Czech Academy of Sciences, České Budějovice 370 05, Czech Republic; Department of Environmental Ecology, Institute of Marine and Environmental Sciences, University of Szczecin, Szczecin 70-383, Poland; Departament de Biologia Evolutiva, Ecologia i Ciències Ambientals, Facultat de Biologia, Universitat de Barcelona (UB), Barcelona 08028, Spain; Institut de Recerca de la Biodiversitat (IRBio), Universitat de Barcelona (UB), Barcelona 08028, Spain; Department of Agronomy, Food, Natural Resources, Animals and Environment, University of Padova, Legnaro 35020, Italy; Institute of Botany, Faculty of Biology, Jagiellonian University, Kraków 30-387, Poland; Institute of Botany, Faculty of Biology, Jagiellonian University, Kraków 30-387, Poland; Institute of Agrophysics, Polish Academy of Sciences, Lublin 20-290, Poland; Department of Plant Taxonomy and Nature Conservation, Faculty of Biology, University of Gdańsk, Gdańsk 80-308, Poland; Institut de Recherche sur la Biologie de l'Insecte, UMR 7261, CNRS – Université de Tours, Tours 37200, France; Department of Biology and Ecology, Faculty of Sciences, University of Novi Sad, Novi Sad 21000, Serbia; Department of Geobotany and Plant Ecology, Faculty of Biology and Environmental Protection, University of Lodz, Lodz 90-237, Poland; Active Learning in Ecology and Biotechnology, Murcia 30149, Spain; Institute of Forest Sciences, INIA-CSIC, Madrid 28040, Spain; Department of Agricultural and Forest Sciences and Engineering, Universitat de Lleida – Agrotecnio CERCA Centre, Lleida 25198, Spain; IMBE, Aix Marseille Université, Avignon Université, UMR CNRS 7263, IRD 237, Marseille, France; INRA Genebank, Regional Center of Agricultural Research of Settat, National Institute of Agricultural Research, Rabat Principale 10090, Morocco; Biome Lab, Department of Biological, Geological and Environmental Sciences, Alma Mater Studiorum - University of Bologna, Bologna 40126, Italy; Faculty of Science, Department of Plant and Environmental Sciences, Section for Plant Biochemistry, University of Copenhagen, Frederiksberg 1871, Denmark; Faculty of Science, Department of Plant and Environmental Sciences, Section for Plant Biochemistry, University of Copenhagen, Frederiksberg 1871, Denmark; Landscape and Conservation Ecology Group, Institute of Ecology and Botany, MTA–HUN-REN Centre for Ecological Research, Vácrátót H-2163, Hungary; Department of Agricultural and Forest Sciences and Engineering, Universitat de Lleida – Agrotecnio CERCA Centre, Lleida 25198, Spain; UK Centre for Ecology & Hydrology, Benson Lane, Oxfordshire OX10 8BB, UK; Department of Plant Biology, Institute of Biology, Wrocław University of Environmental and Life Sciences, Wrocław PL-51-631, Poland; Biome Lab, Department of Biological, Geological and Environmental Sciences, Alma Mater Studiorum - University of Bologna, Bologna 40126, Italy; Institute for Plant Sciences, Cluster of Excellence on Plant Sciences (CEPLAS), University of Cologne, Cologne 50674, Germany; Cluster of Excellence on Plant Sciences ‘SMART Plants for Tomorrow's Needs’, Düsseldorf 40223, Germany; Institute of Plant Genetics, Department of Biology, Heinrich-Heine-University Düsseldorf, Düsseldorf 40225, Germany; Cluster of Excellence on Plant Sciences ‘SMART Plants for Tomorrow's Needs’, Düsseldorf 40223, Germany; Conservation Biology, Department of Biology, Philipps University Marburg, Marburg 35043, Germany

**Keywords:** Climate, environment, grasses, *Hordeum murinum*, *in situ* sampling, intraspecific trait variability, ruderals, seed elemental nutrients

## Abstract

**Background and Aims:**

Plant functional traits link environmental conditions to plant performance and adaptation. Growing evidence suggests that intraspecific trait variation can be as important as differences between species, yet large intraspecific studies of *in situ* variation remain rare. Most studies have focused on plant morphological traits, whereas the concentrations of elemental nutrients in seeds have received much less attention so far.

**Methods:**

We conducted a large-scale *in situ* study of the widespread annual ruderal grass *Hordeum murinum*. We sampled 2070 individuals from 207 populations across a large part of its native range in Europe and North Africa. We measured seed ripening phenology and growth-related traits *in situ* and analysed concentrations of elemental nutrients in the seeds.

**Key Results:**

We found that *H. murinum* grew larger, produced seeds later and had heavier seeds in colder and wetter regions. Plants growing in denser vegetation were taller and produced heavier seeds but formed fewer spikes. Concentrations of elemental nutrients in the seed generally declined with seed weight and were driven primarily by climatic variables, whereas soil conditions had only minor effects on plant traits and seed nutrients. Population identity explained a substantial proportion of trait variation, indicating a possible genetic component.

**Conclusions:**

Our findings provide a comprehensive view of how *H. murinum* responds to environmental gradients across its European distribution. Climatic variables, particularly temperature, are key drivers of reproductive timing and concentrations of elemental nutrients in the seed, whereas local environmental conditions, such as biotic pressures, are more important for growth-related traits. Together, these patterns indicate that *H. murinum* modulates its growth and reproductive investment along environmental gradients, balancing phenology, stress tolerance and limited competitive capacity.

## INTRODUCTION

Plant functional traits are key adaptations to optimize plant performance depending on environmental conditions (Bruelheide *et al.*, 2018). Although most studies have focused on comparing mean functional traits across species or entire communities (Moles *et al.*, 2009), substantial evidence documents considerable intraspecific variation in plant functional traits (De Frenne *et al.*, 2013; Albert, 2015). This variation reflects functional differences within and among plant populations in response to local environmental conditions (Klein-Raufhake *et al.*, 2022), and its magnitude can be similar to variation observed between species (Tautenhahn *et al.*, 2019). Heritable intraspecific trait variation is typically evaluated in common garden experiments (Leiblein-Wild and Tackenberg, 2014), but trait measurements in natural environments are essential for an ecologically meaningful understanding of how individuals perform in such conditions (Helsen *et al.*, 2017).

Key life-history and morphological traits, such as reproductive phenology, plant height, specific leaf area and seed weight, directly influence plant survival and reproductive success (Farris and Lechowicz, 1990; Adler *et al.*, 2014). Variation in these traits reflects local abiotic and biotic conditions, including water availability, soil nutrients, pH and intensity of competition (Lechowicz and Blais, 1988; Andrade *et al.*, 2014; Helsen *et al.*, 2017). Life-history and morphological traits also vary along large-scale climatic gradients in temperature and precipitation (Leiblein-Wild and Tackenberg, 2014; Lemke *et al.*, 2015). Annual plants growing in warmer and drier climates often flower earlier than their conspecifics growing in colder and wetter climates, which allows them to complete their life cycles before environmental conditions become too dry and hot during summer (Evans *et al.*, 2005). Plants in drier regions are generally shorter and produce fewer spikes than conspecifics in wetter regions, a pattern commonly attributed to adaptation to water limitation and thermal stress (Nour *et al.*, 2024). Based on a meta-analysis, De Frenne *et al.* (2013) found that plants from low-latitude populations tend to produce heavier seeds than conspecific individuals from high-latitude populations, although the direction and magnitude of latitude effects differ considerably among species. Nevertheless, studies of intraspecific variability in life-history and morphological traits are still limited, particularly in sufficiently high spatial resolution across large geographical and climatic gradients.

An important but often overlooked set of plant traits is the concentration of elemental nutrients in the seeds. They are essential for seedling establishment and early survival, because seedlings initially rely entirely on internal nutrient reserves before root systems are sufficiently developed for external acquisition (Slot *et al.*, 2013). Like morphological plant traits, seed nutrient concentration is affected by the environment. Nutrient-rich soils generally promote higher seed nutrient concentrations, as greater soil nutrient availability increases the amount of nutrients available for seed filling (Long *et al.*, 2025). Climatic factors such as temperature and water availability can modulate seed nutrient concentrations further, partly through their effects on nutrient uptake and the duration of seed filling (Sehgal *et al.*, 2018). Seed nutrient concentrations also vary along environmental gradients, with the direction of these relationships being species specific (Etienne *et al.*, 2018). Most of our understanding of how the environment affects variation in seed nutrients comes from crop species, and there is a notable gap regarding the intraspecific variability in the concentration of elemental nutrients in seeds in wild plants (see De Frenne *et al.*, 2011; Li *et al.*, 2023; Wu *et al.*, 2024; Wang *et al.*, 2025).

Plant functional traits are not independent, but often covary within species (Sandel *et al.*, 2016). For example, larger plants often produce larger seeds (Hendrix and Sun, 1989; Guo *et al.*, 2010; He *et al.*, 2023). Concentrations of seed elemental nutrients tend to decrease with greater seed weight (Uauy *et al.*, 2006), probably because larger seeds contain a higher proportion of carbohydrates or lipids, hence lower concentrations of elemental nutrients (Wang *et al.*, 2016). However, empirical data examining intraspecific covariation between seed nutrient concentration and morphological traits in populations of wild species growing in their characteristic habitats remain scarce (Henery and Westoby, 2001; De Frenne *et al.*, 2011).

*In situ* intraspecific trait variation in wild plants is particularly interesting in species with broad geographical distributions. These species encounter a wide range of environmental conditions, which is reflected in a large variation in life-history traits (Lemke *et al.*, 2015). Such variation can be related to, for example, climatic, edaphic or land-use gradients. However, such gradients often covary across large scales, which makes the identification of the drivers of intraspecific variability based on a small number of populations difficult (De Frenne *et al.*, 2013; Helsen *et al.*, 2017). Disentangling the effects of individual environmental factors on intraspecific variation requires data collected from many populations across diverse environments, at high spatial resolution and on a large scale, but such data are rare so far.

To fill this gap, we focused on *in situ* trait–environmental association of a wild grass, *Hordeum murinum* L. (mouse barley, wall barley), a relative of cultivated barley (*Hordeum vulgare* L.). This species is native to Europe and adjacent regions, widespread, and commonly occurs in human-disturbed habitats (Davison, 1977). We measured seed ripening phenology and growth-related traits of 2070 plants from 207 populations distributed from North Africa to southern Scandinavia. For a subset of 940 plants from 196 populations, we additionally quantified the concentration of elemental nutrients in the seeds. We related these traits to climatic variables, local competition intensity and soil conditions.

Specifically, we tested the following three main hypotheses:

1. Seed ripening phenology, growth-related traits and concentrations of elemental nutrients in the seeds covary with each other. Specifically, we expect that growth-related traits will be negatively correlated with seed ripening phenology, because plants that grow longer and larger invest in reproduction only later. Growth-related traits will also be negatively correlated with concentrations of elemental nutrients in the seed, because larger plants produce larger seeds that contain more carbohydrates, hence lower concentrations of elemental nutrients through a dilution effect.
2. Seed ripening phenology and growth-related traits will vary mainly along climatic gradients. Specifically, we expect that plants in warmer and drier areas will be smaller and reproduce earlier because they prioritize reproduction over growth in spring to avoid heat and drought stress in summer. Plants in colder and wetter areas can grow longer and larger and can reproduce later.
3. Concentrations of elemental nutrients in the seeds will be associated mainly with local conditions, particularly soil quality, because plants growing in nutrient-rich soils have access to more nutrients to invest in the seeds.

## MATERIALS AND METHODS

### Study species

*Hordeum murinum* L. is a winter annual grass native to the Mediterranean region, most of Europe, Central Asia, Western Himalayas, Macaronesia and the Azores. It has been introduced by humans to Australia, North America and many other parts of the world, where it has become invasive (Jacobsen and von Bothmer, 1995; Fleet and Gill, 2010). Across most of its range, *H. murinum* typically grows in regularly disturbed open habitats, such as fallow land, roadsides, urban green spaces (e.g. median strips), riverbanks, vineyards, agricultural field margins and, only rarely, in meadows (Davison, 1977).

*Hordeum murinum* typically flowers between early spring and late summer, depending on the region. Flowers are predominantly self-pollinating, and seeds are dispersed through animals or humans when spikelets adhere to animal fur or human clothing. Seeds typically germinate from late summer or early autumn through winter; plants overwinter in a vegetative stage and start flowering in spring (Davison, 1977; Jacobsen and von Bothmer, 1995; Mizianty, 2006).

*Hordeum murinum* is a part of an aggregate taxon *Hordeum murinum* agg. that comprises three subspecies differing by cytotypes: diploid [2*n* = 2*x* = 14, subspecies *glaucum* (Steud.) Tzvelev]; tetraploid (2*n* = 4*x* = 28, subsp. *murinum* or *H. murinum s.s.*); and hexaploid [2*n* = 6*x* = 42, subsp. *leporinum* (Link)] (Cuadrado *et al.*, 2013). This study focuses on the tetraploid cytotype, *H. murinum* subsp. *murinum*, across Europe and the Mediterranean region (Fig. 1). In the southern part of this region, below latitude 45°, *H. murinum* also occurs as diploid (Jacobsen and von Bothmer, 1995), but the exact distribution of the two cytotypes is unclear. Given that we could not differentiate the subspecies in the field based on morphology, we determined the cytotype of the sampled populations via flow cytometry (see Appendix). Tetraploid populations were detected throughout the entire sampled range, whereas both diploids and tetraploid populations occurred in mainland Spain, Algeria and Morocco, and some populations contained both ploidy levels (Supplementary Data Fig. S1). Given that we focused on tetraploids here, we excluded all diploid and mixed populations from the main analyses. Differences between populations of diploids and tetraploids are not addressed here, but corresponding data are provided in the Appendix to document cytotypic variation within the sampled dataset (Supplementary Data Figs S1–S3; Table S1).

**Fig. 1.**
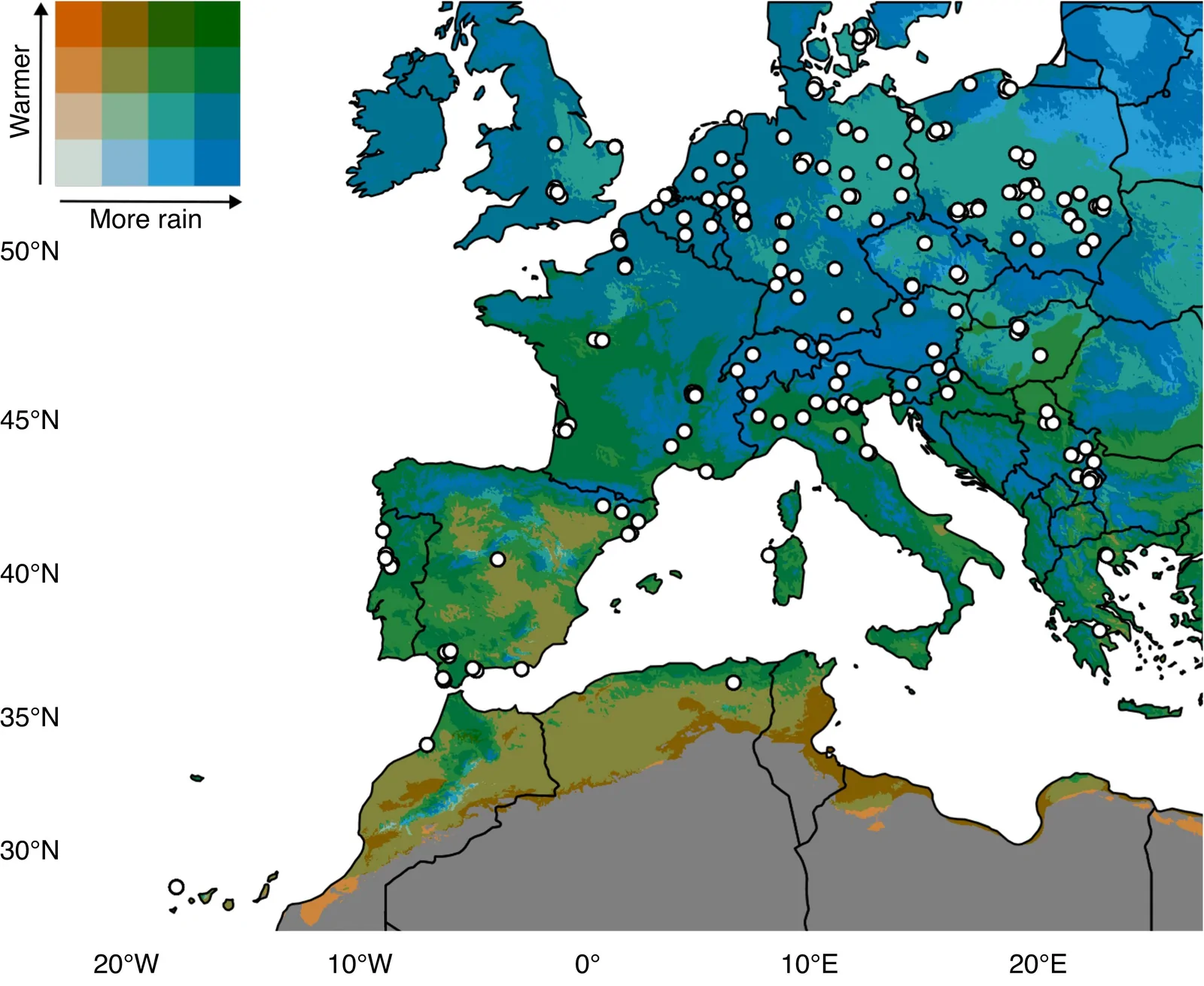
Climate map of the *in situ* sampled populations of *Hordeum murinum* across Europe and North Africa. Colour shading shows the average climate from the years 2000–2020 of mean annual temperature and annual precipitation. Only tetraploid populations are shown (for diploids and mixed populations, see Supplementary Data Fig. S1).

### Plant material and *in situ* trait scoring

We scored and sampled 242 natural populations of *H. murinum* across Europe, of which 207 populations were identified as exclusively tetraploid and included in the main analyses (Fig. 1). Most populations were sampled during the seed ripening period in summer 2023, whereas six populations were sampled in 2022 and four in 2024. Depending on geographical location, the seed ripening period ranged between March and August. We defined a population as a site with the presence of at least ten individual plants. We kept ≥1 km distance between two neighbouring populations, with a median nearest-neighbour distance of ∼18 km, to increase the likelihood that the populations are genetically differentiated.

Within each population, we randomly sampled ten plants spaced ≥5 m apart. For each plant, we measured plant height, defined as the length of the longest tiller excluding the spike, because ripe spikes tended to disintegrate as soon as we touched the plant. We then recorded the number of tillers bearing spikes (both unripe and ripe) and harvested one spike per plant with ≥50 % visibly ripe seeds. In the laboratory, we extracted all seeds from the harvested spikes, counted and weighed them, and stored them in a refrigerator. Given that the spikes rapidly disintegrate when ripe, we rarely collected a whole spike. We thus did not use the number of seeds per spike in further analysis, but rather seed weight standardized to thousand-seed weight.

We used the seed collection date as a proxy for seed ripening phenology. We mostly sampled the seeds when we observed that the first spikes in a population were yellow, indicating ripe seeds. In some cases, later collection was unavoidable; however, plants typically retain ripe seeds for ∼2–3 weeks before complete seed shattering. This introduces only limited uncertainty at the population level relative to inter-populational differences, which spanned ∼6 months.

For 6 of the 207 tetraploid populations, all spikes were empty, without seeds; however, seed ripening phenology, growth-related traits and environmental measurements were still available. For two populations, we obtained ripe spikes and soil samples (see below), but measurements of growth-related traits and local environmental variables were missing. These populations were retained where possible for analyses not requiring the missing variables.

### Environmental variables

To characterize local environmental conditions, we focused on the intensity of competition and soil characteristics. We visually estimated vegetation cover and the cover of impervious surfaces (e.g. asphalt, cobblestone or concrete) within a 1 m^2^ area surrounding each sampled plant. We also collected soil samples from beneath each sampled plant and pooled at the population level. Soil samples were air-dried at room temperature and stored in refrigerated conditions upon arrival at the laboratory.

We obtained climatic variables from the CHELSA database (Karger *et al.*, 2017), which provides climate data for the period 2000–2020 at ∼1 km^2^ (30 arcsec) spatial resolution. We initially extracted all ten bioclimatic variables that were available at the 30 arcsec resolution. Because these variables were highly collinear (*r* ≥ |0.5|; Supplementary Data Fig. S4), we retained only mean annual temperature (in degrees Celsius), temperature seasonality (in degrees Celsius, s.d. of the monthly mean temperatures) and annual precipitation (in millimetres) for subsequent analyses, because they allow intuitive interpretation and were the least correlated with other variables.

To measure the pH of the soil, we added 25 mL of 0.01 M CaCl_2_ solution to a subsample of 10 g of the pooled soil samples, followed by shaking for 1 h. After the suspension had settled, we measured the pH of the supernatant using a calibrated pH electrode (HANNA instruments, pH 211 Microprocessor pH Meter).

To quantify plant-available inorganic nitrogen, phosphorus and sulphur, as a proxy for readily available soil nutrients in the soil, we analysed soil concentrations of nitrate, phosphate and sulphate. Specifically, 100 mg of soil was extracted in 300 μL of deionized water for 6 h at 30 °C. After 15 min centrifugation at 4 °C at a maximum speed of 12 000*g*, the supernatant was transferred into HPLC vials with inlets. Inorganic anions were quantified using a Dionex ICS-1100 ion chromatography system equipped with a Dionex IonPac AS22 RFIC (4 mm × 250 mm) analytical column (Thermo Scientific, Darmstadt, Germany, Dietzen et al., 2020). A 4.5 mM NaCO_3_, 1.4 mM NaHCO_3_ solution was used as a running buffer. Standard curves were generated using the external standard solutions of 0.05, 0.1, 0.2 and 0.5 mM KNO_3_, K_2_HPO_4_, K_2_SO_4_ and KCl, respectively.

### Concentration of elemental nutrients in the seed

We used inductively coupled plasma–mass spectrometry (ICP-MS) to determine the elemental concentrations of nutrients in the seed, specifically macronutrients Ca, K, Mg, P and S and micronutrients Cu, Fe, Mn, Mo, Ni and Zn (Almario *et al.*, 2017, Huppertz et al., 2026). Two to three seeds per individual (8–20 mg) were dried at 60 °C overnight and homogenized to a fine powder. The seed material was digested with 500 μL of 67 % (w/w) HNO_3_ in 15 mL polypropylene Falcon tubes overnight at room temperature. Subsequently, loosely closed tubes containing the samples were heated in a 95 °C water bath until the liquid became completely clear (∼30 min). After cooling to room temperature for 10–15 min, the samples were placed on ice and diluted with 4.5 mL of deionized water. The samples were centrifuged at 2000*g* for 30 min at 4 °C, and the supernatants were transferred into new tubes for analysis. The elemental concentrations were determined using an Agilent 7700 ICP-MS (Agilent Technologies) (Almario *et al.*, 2017). The selected method allows quantification of many elemental nutrients except nitrogen, because it involves digesting the material in HNO_3_. We acknowledge that nitrogen is important for plant performance, and the absence of data on this element is a limitation of this study. Future studies combining elemental analysis with nitrogen quantification methods would provide a more complete picture of seed nutrient composition and its ecological relevance.

To reduce analytical costs while maintaining broad geographical coverage, we analysed the concentration of nutrients in the seeds for only a subset of six randomly selected plants per population and restricted sampling to populations located ≥10 km apart. This design aimed to capture large-scale spatial variation in seed nutrient concentrations without compromising the assessment of fine-scale variability. In total, we analysed the concentrations of elemental nutrients in the seed for 940 individuals from 196 populations.

### Statistical analysis

First, we explored the strength and direction of covariation among seed ripening onset, growth-related traits (plant height, seed weight, and number of spikes) and concentrations of elemental nutrients in the seed. We performed Pearson’s correlation analyses and visualized significant correlations using a correlation matrix generated with the *Hmisc* package (Harrell Jr, 2025). Because seed ripening onset was available only at the population level, we used the population means for correlations involving this trait. Correlations among other traits were calculated at the individual plant level. All correlation tests were corrected for multiple testing using Benjamini–Hochberg correction (Benjamini and Hochberg, 1995).

We applied the same approach to examine correlations among environmental predictors, including the selected climatic variables, vegetation cover, impervious surface cover and soil properties. For this analysis, we used population-level averages for all predictors because most environmental variables were measured at the population level, except vegetation cover and impervious surface cover. Vegetation cover and impervious surface cover showed a strongly negative correlation (*r* = −0.62), which adversely affected model stability. We therefore retained only vegetation cover for subsequent analyses.

Second, we assessed whether populations differ in terms of growth-related traits and concentrations of elemental nutrients in the seed. To do this, we ran separate linear models for each parameter, using population identity as a categorical predictor (Supplementary Data Table S2). Seed ripening onset could not be included in this analysis because it was available only at the population level.

Third, we tested how the onset of seed ripening, growth-related traits and concentrations of elemental nutrients in the seed relate to climate and environmental factors. Explanatory variables were three climatic predictors (mean annual temperature, temperature seasonality and annual precipitation) and five local environmental variables (vegetation cover, soil pH and bioavailable soil nutrients: nitrate, phosphate and sulphate).

The onset of seed ripening was available only at the population level. We thus related the onset of seed ripening to population-level averages of the environmental variables in a linear model. To test for spatial autocorrelation, we applied the function *moran.test*, in the *spdep* package (Bivand, 2022), to the model and found significant spatial autocorrelation in the ordinary least-squares model residuals (Moran’s *I* = 0.12, *P* < 0.001). Therefore, we refitted the model using a spatial lag regression, *spatialreg* package (Bivand *et al.*, 2021), which substantially reduced spatial autocorrelation in the residuals (Moran’s *I* = 0.04, *P* = 0.08). Because spatial regression models do not provide conventional *R*^2^ values, we quantified model fit using a pseudo-*R*^2^ statistic, calculated as one minus the ratio of the residual variance to the total variance of the response variable (Veall and Zimmermann, 1996). To assess the relative contribution of individual predictors, we compared changes in the Akaike information criterion (AIC) after sequentially removing each predictor from the full model (Burnham and Anderson, 2002). The resulting ΔAIC values were standardized to percentages to express the relative contribution of each predictor to overall model fit.

Growth-related traits and concentrations of elemental nutrients in the seed were available at the level of individual plants. We thus built 14 linear mixed models (using the *lme4* package; Bates *et al.*, 2015), one model for each of the 3 plant growth-related traits and the 11 investigated seed nutrients as respective response variables. As fixed explanatory variables, we used the three climatic and five local environmental predictors. Population identity was included as a random effect to account for the non-independence of plants within each population. To test for spatial autocorrelation, we plotted the residuals from each of the 14 linear mixed models on a map, with point size representing residual magnitude and colour indicating whether the residuals were negative or positive. We did not find discernible spatial patterns that would indicate autocorrelation of the residuals and retained the original models (Dale and Fortin, 2014). To determine the proportion of variation explained by the models (fixed, random and whole model *R*^2^ value), we used the *rsq* package (Zhang, 2016). The relative importance of individual fixed-effects predictors was assessed using the *partR2()* function from the *partR2* package (Stoffel *et al.*, 2021).

To meet model assumptions, we log- or square root-transformed the response variables where necessary (Supplementary Data Tables S3–S5) and evaluated the model fit through residual diagnostics (Zuur *et al.*, 2010). All data analyses were conducted using R v.4.3.1 (R Core Team, 2023) within the RStudio environment.

## RESULTS

The onset of seed ripening, an aspect of plant reproductive phenology, was positively correlated with the average number of spikes per plant and negatively correlated with concentrations of elemental nutrients in the seeds (Fig. 2). In other words, populations that ripened seeds later tended to produce more spikes per plant, whereas their seeds had lower concentrations of elemental nutrients. Growth-related traits (plant height, seed weight, and number of spikes) were positively correlated with each other, with the strongest relationship between plant height and seed weight (*r* = 0.60; Fig. 2). Likewise, ∼70 % of the pairwise correlations among seed nutrient concentrations were positive. The strongest positive correlation was between P and Mg (*r* = 0.65). Seed ripening onset and growth-related traits generally exhibited negative correlations with elemental nutrient concentrations in the seeds, particularly Ca, K, Mg, P, Ni and Zn. The strongest negative correlations were between seed ripening onset and K (*r* = −0.49) and between seed weight and Ca and K (both *r* = −0.44). Molybdenum (Mo) deviated from this pattern, showing a positive correlation with seed ripening onset (*r* = 0.31), seed weight and number of spikes (both *r* = 0.09). Pairwise correlations between environmental predictors (climate, ground cover and soil properties), even when significant, were weak (in most cases *r* < 0.3; Fig. 3). The only exceptions were correlations between vegetation cover and cover of impervious surface (*r* = −0.62) and between mean annual temperature and temperature seasonality (*r* = −0.44; Fig. 3).

**Fig. 2.**
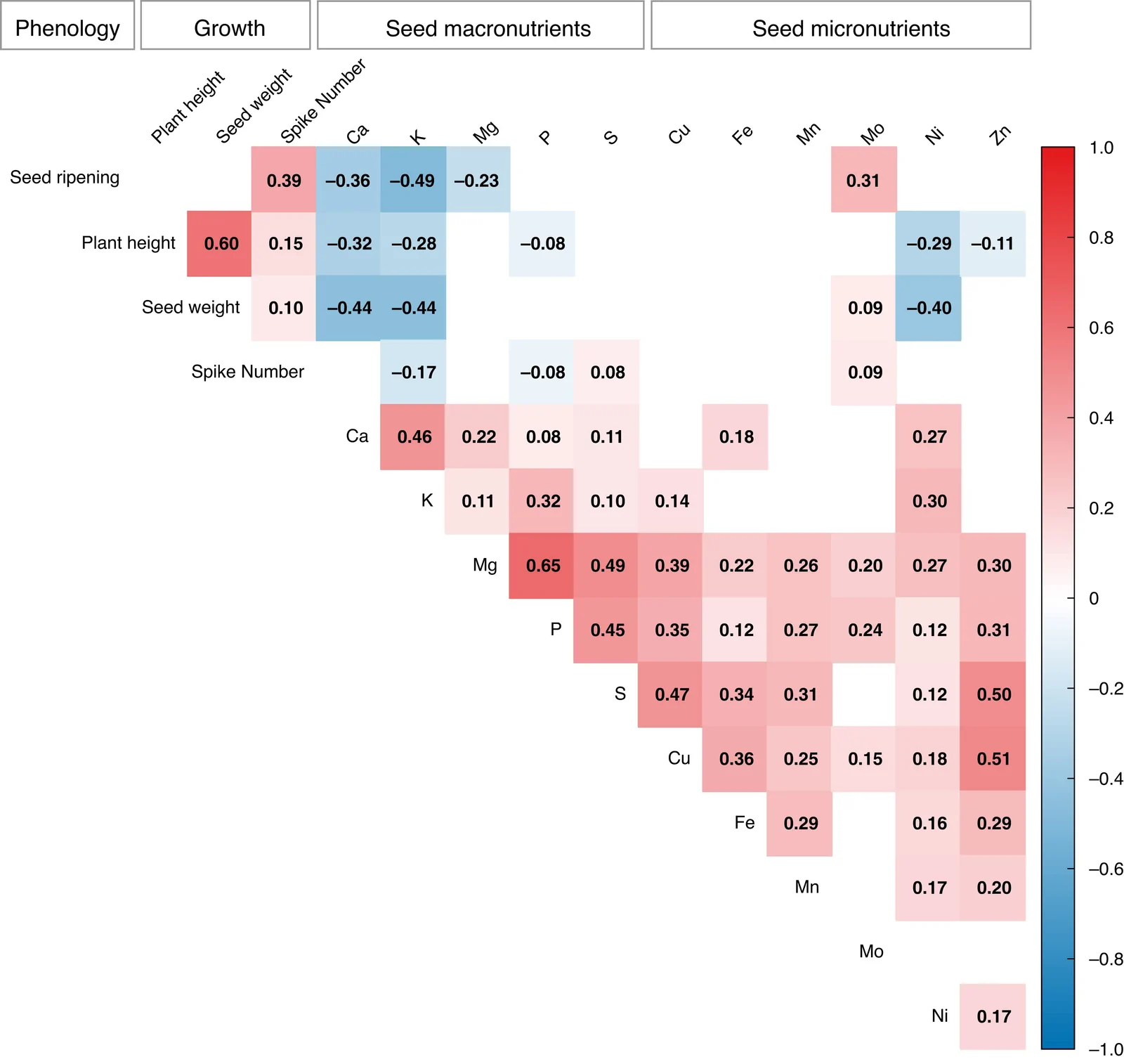
Pearson's correlation coefficient between seed ripening phenology, growth-related traits and seed macro- and micronutrient concentrations. The number and colour intensity in the cells indicate the strength and direction of the individual correlation. Empty cells indicate non-significant relations (*P* > 0.05). The significance threshold was adjusted for multiple testing using Benjamini–Hochberg correction.

**Fig. 3.**
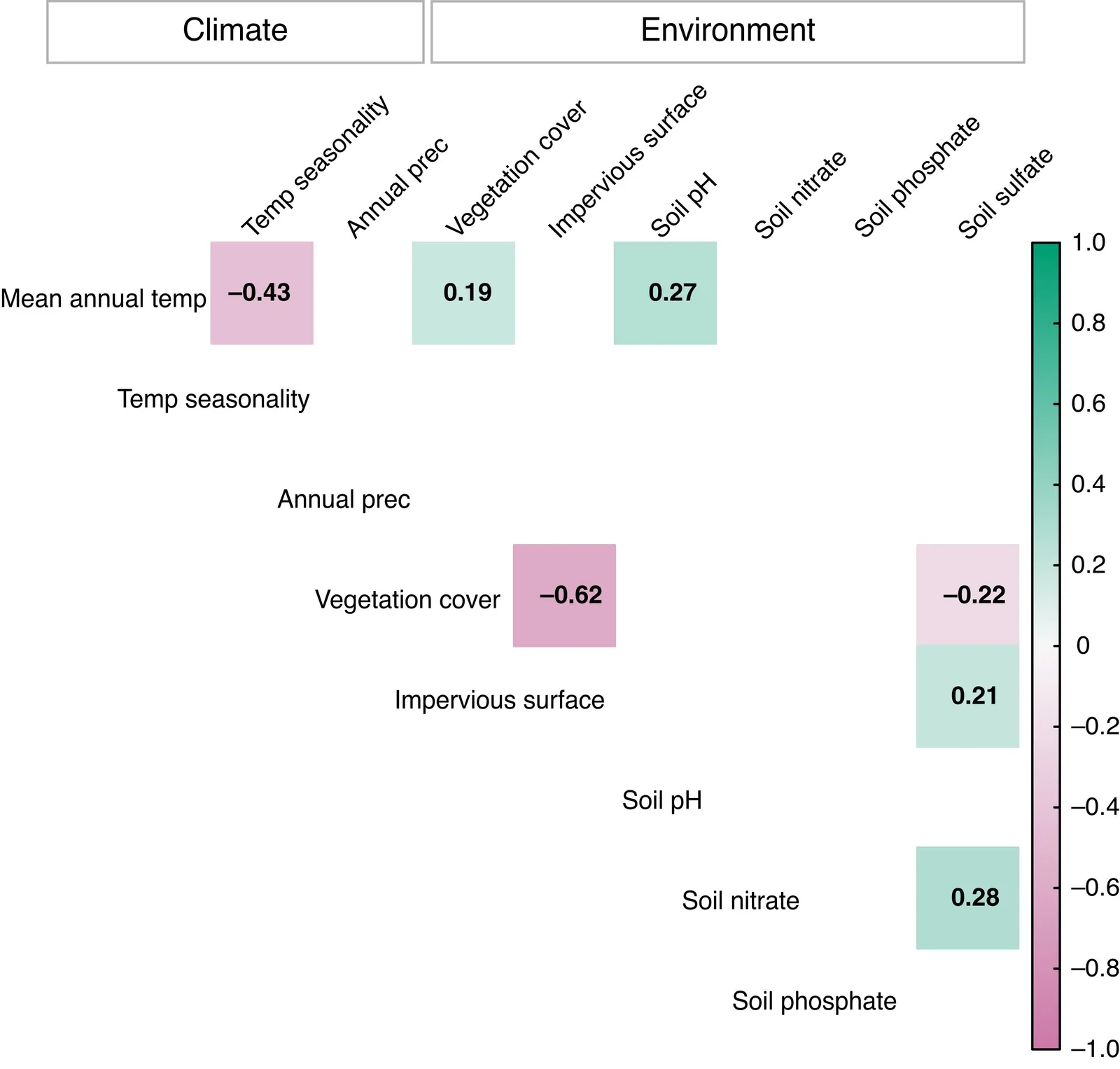
Pearson’s correlation coefficient between all predictors. Note that most predictors were available at population level only, and vegetation and impervious surface were averaged across all plants within each population. The number and colour intensity in the cells indicate the strength and direction of the individual correlation. Empty cells indicate non-significant relations (*P* > 0.05). The significance threshold was adjusted for multiple testing using Benjamini–Hochberg correction.

All three growth-related traits were significantly differentiated among populations, with population identity explaining 37–38 % of the variation when we fitted population identity as a single predictor (Supplementary Data Table S2). Seed nutrient concentrations were also significantly differentiated among populations, with the highest differentiation observed for Cu (*R*^2^ = 0.57) and the lowest for Ni (*R*^2^ = 0.24). In models including environmental predictors as fixed variables and population identity as random factor, population identity (marginal *R*^2^) still explained more variability than the environmental predictors in both growth-related traits and seed nutrient concentrations (pie charts in Figs 4 and 5; Supplementary Data Tables S2–S4). Population differentiation in seed ripening onset could not be assessed because this trait was represented by a single value per population.

**Fig. 4.**
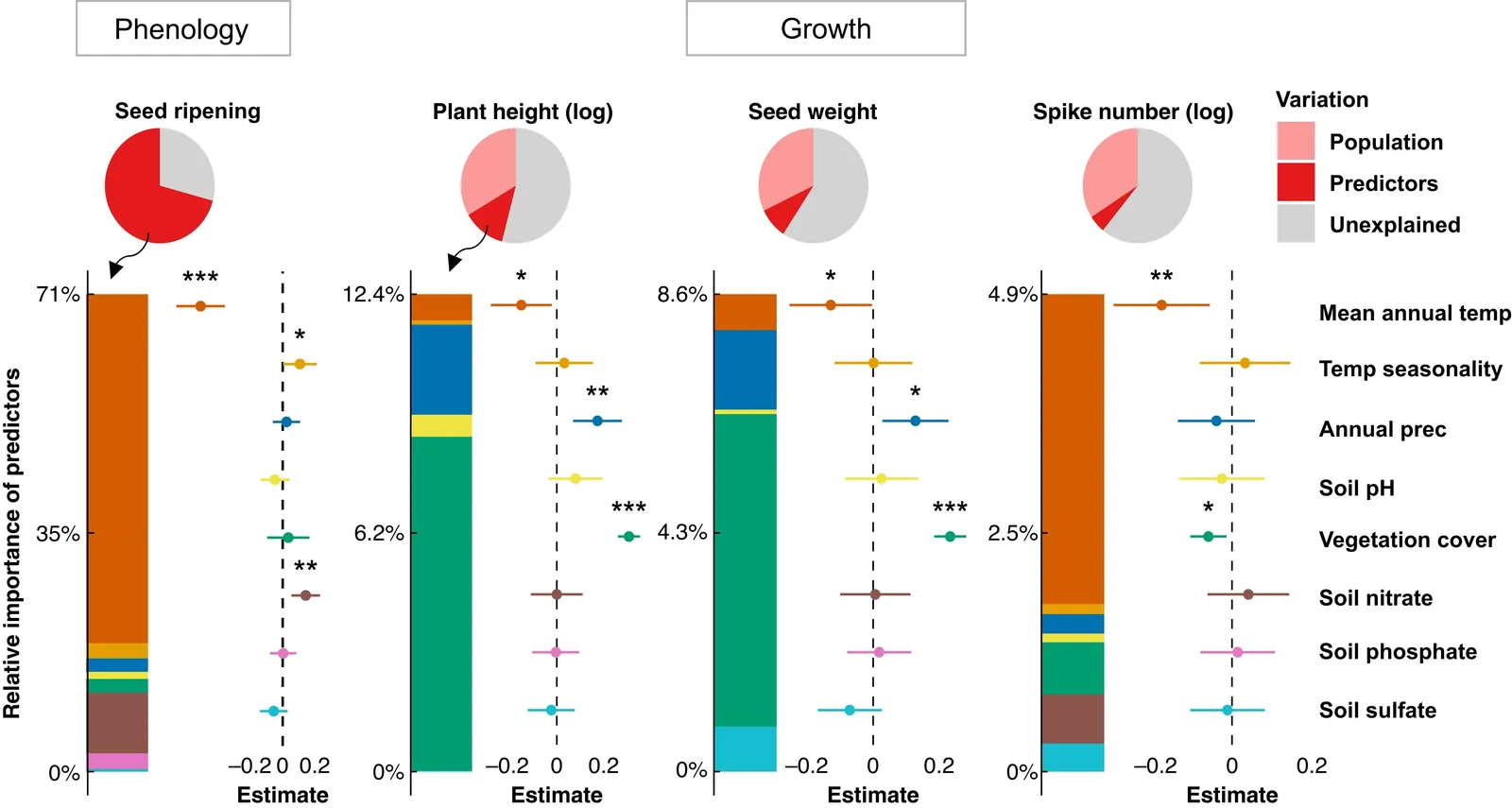
Intraspecific variation in plant phenology and growth-related traits. Results of a spatial lag model and a linear mixed model, respectively, testing for the effect of climate and environment. Each plot contains three main components, namely pie charts, bar plots and forest plots. Pie charts show the *R*^2^ partitioning of each model split into population (variance explained by the random effect, light red), predictors (variance explained by the fixed effects, dark red) and unexplained variance (grey). Bar plots show the estimated relative importance of the environmental predictors (proportion of explained variance of each predictor). Forest plots exhibit effect size estimates (points) with confidence intervals (error bars). Asterisks indicate the significance levels of each predictor (*P < 0.05, **P < 0.01 and ***P < 0.001). Predictors were scaled before the analysis. Some response variables were log-transformed before analysis (as indicated in the variable name).

**Fig. 5.**
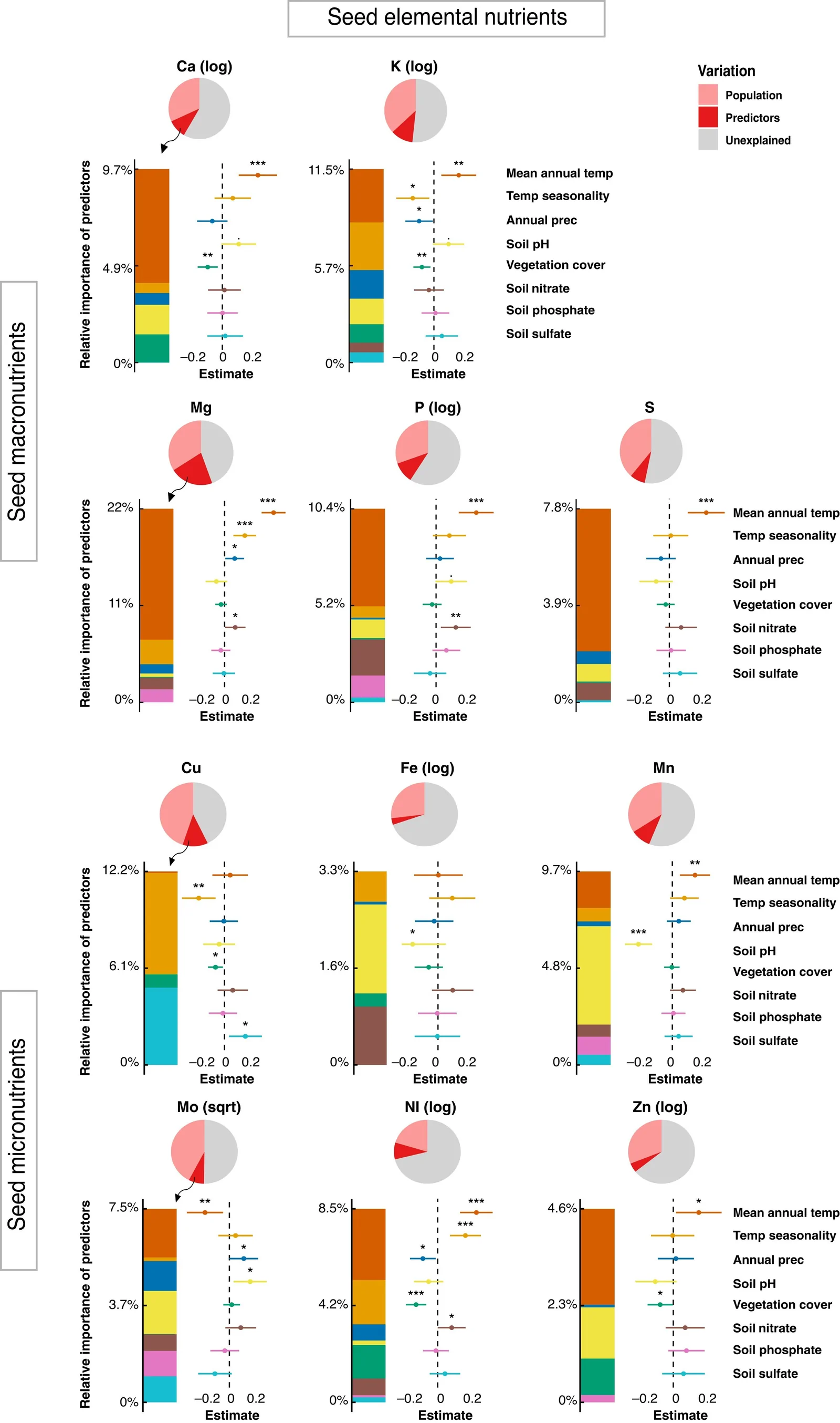
Intraspecific variation in concentrations of elemental nutrients in the seed; results of linear mixed model testing for the effect of climate and environment. Each plot contains three main components, namely pie charts, bar plots and forest plots. Pie charts show the *R*^2^ partitioning of each model split into population (explained variance by the random effect, light red), predictors (explained variance by the fixed effects, dark red) and unexplained variance (grey). Bar plots show the estimated relative importance of the environmental predictors (proportion of explained variance of each predictor). Forest plots exhibit effect size estimates (points) with confidence intervals (error bars). Asterisks indicate the significance levels of each predictor (*P < 0.05, **P < 0.01 and ***P < 0.001). Predictors were scaled before the analysis. Some response variables were log-transformed before analysis (as indicated in the variable name).

Seed ripening phenology was related primarily to climate and, to a lesser extent, soil nitrate. Seed ripening occurred earlier in populations in warmer regions and in regions with lower temperature seasonality. Together, these two climatic variables explained ∼88 % of the variation in seed collection timing among populations. Seed ripening occurred slightly later in nitrate-rich soils, although this variable explained only a small proportion of variation (*R*^2^ = 0.04).

Growth-related traits were related to local vegetation cover and climate. Plant height and seed weight were affected primarily by vegetation cover, annual precipitation and mean annual temperature, with increased vegetation cover, higher annual precipitation and lower mean annual temperature leading to taller plants and heavier seeds (Fig. 4). The number of spikes was most affected by mean annual temperature and vegetation cover, with the number of spikes per plant being higher in colder climates and when plants were surrounded by less vegetation.

The concentrations of elemental nutrients in seeds (Ca, K, Mg, P, S, Mn, Mo, Ni and Zn) were most affected by the mean annual temperature. In general, warmer climates were associated with higher elemental concentrations, except for Mo, which showed an opposite response (Fig. 5). Effects of temperature seasonality and annual precipitation varied among nutrients; for example, K and Cu decreased with increasing seasonality, whereas Mg and Ni increased with temperature seasonality. Local predictors also showed element-specific effects. For example, seed Fe and Mn concentrations decreased significantly with soil pH, whereas Mo increased with soil pH. Nitrate had a positive effect on the concentration of Mg, P and Ni in seeds. Other soil properties, such as phosphate and sulphate levels, mostly had no effect on the seed elemental nutrient concentration, except for soil sulphate, which had a positive effect on seed Cu. Vegetation cover was negatively associated with Ca, K, Zn, Cu and Ni, although the variance explained by vegetation cover was low (Fig. 5).

## DISCUSSION

Understanding how plants respond to environmental change requires integrative analyses linking plant functional traits with local environmental conditions and broad-scale climatic gradients (Moran *et al.*, 2016). Here, we present a comprehensive, continental-scale analysis of intraspecific trait variation in a wild plant species spanning ∼4500 km. The dataset combines *in situ* seed ripening phenology and growth-related traits of 2070 individual plants across 207 populations, complemented by concentrations of elemental nutrients in the seeds for a subset of 940 individuals of *H. murinum*. To our knowledge, this dataset represents one of the most extensive collections of *in situ* data on seed elemental nutrient concentrations in a wild plant species across such a broad geographical gradient. This unique dataset allowed us to determine that variations in reproductive phenology, growth-related traits and seed nutrient concentrations are not driven by the same environmental factors.

Our results reveal clear ecological patterns: *H. murinum* in colder and wetter regions grew larger, produced seeds later and had heavier seeds. Plants growing in denser vegetation were also taller and produced heavier seeds but formed fewer spikes, highlighting the importance of local biotic context for growth-related traits. Seed elemental concentrations tended to decline with increasing seed weight and were associated primarily with climatic variables, whereas soil properties generally showed weak associations with both growth-related traits and concentrations of elemental nutrients in the seed. Importantly, population identity explained a substantial proportion of variation in plant traits and concentrations of elemental nutrients in the seed beyond the effects of measured environmental predictors, suggesting the presence of persistent population-level differences.

### Plant phenology and growth-related traits

Plants in colder regions ripened seeds later in the season. Specifically, seed collection dates, used as a proxy for seed ripening phenology, ranged from March in Mediterranean regions to August in northern Central Europe. Temperature effects on plant phenology are widespread across plant species (Boyko *et al.*, 2023), particularly in annuals, which often complete their life cycle earlier in spring in warm and dry regions to avoid summer drought and heat stress (Evans *et al.*, 2005). Our results are therefore in line with these general patterns.

Plants were generally larger in colder and wetter regions. Specifically, plant height, seed weight and the number of spikes were all positively intercorrelated; they decreased with mean annual temperature and increased with mean annual precipitation (significant for the first two traits). A similar increase in plant height in colder areas has been reported by De Frenne *et al.* (2011), who attributed this to the longer photoperiod during the growth season in northern (colder, longer photoperiod during growing season) versus southern (warmer, shorter photoperiod) parts of Europe. In the case of *H. murinum*, plants in the warmer and drier regions in the south senesce in spring to avoid losses through drought and heat stress, which means that they experience a relatively short growing period and grow during winter, when the photoperiod is relatively short. In contrast, plants in the colder and wetter regions can prolong their growth into late spring, when the photoperiod is long; they accumulate more biomass and, consequently, more carbohydrates in the seeds (Dupont and Altenbach, 2003).

Plant height and seed weight also increased in denser vegetation (Fig. 4). Plants commonly grow taller in denser vegetation to escape competition for light (Moles *et al.*, 2009), and plant size shows a positive genetic correlation with seed weight in barley (He *et al.*, 2023). Dense vegetation also slightly reduced the number of spikes produced per plant, probably because of increased competition, with plants having to invest more in elongation growth to escape competition for light, rather than investing in reproduction. Plants in denser vegetation thus invested their carbohydrates in fewer seeds, leading to increased individual seed weight (Salisbury, 1942). It is also possible that denser vegetation implies more resources that can be invested in the seed, but vegetation cover was not correlated with precipitation as a proxy of water availability, nor with nutrient availability in the soil, except for sulphate, but even this relationship was weak.

Available soil nutrients and soil pH had no significant effect on the growth-related traits and had only a limited effect on plant phenology. This is surprising given the ample literature documenting the effect of nutrient addition on traits such as height or number of flowers (Lechowicz and Blais, 1988; Andrade *et al.*, 2014; Barker and Pilbeam, 2015). However, increased soil nutrient availability might promote the growth of co-occurring species, thereby intensifying competitive pressure and potentially negating any specific growth benefits to individual plants (Tan *et al.*, 2025). Furthermore, some nutrients, particularly nitrate and sulphate, are highly mobile in soil and prone to leaching, and concentrations of these nutrients in soil at the time of sampling possibly do not reflect the availability of these nutrients during the mother plant growth and seed development (Bünemann and Condron, 2007; Cameron *et al.*, 2013). Another possible reason might lie in our sampling design, in that we used one bulk soil sample per population. If the soil conditions of individual plants within the site varied strongly, we did not capture this variation, which might have reduced our ability to detect the effect of soil on plant performance. Nonetheless, our results correspond to recent analyses of global intraspecific trait variability in grasses, which have found that soil characteristics are far less important than climate (Griffin-Nolan *et al.*, 2025).

### Concentrations of elemental nutrients in the seeds

Our dataset, comprising seeds from almost 1000 plants chemically analysed for concentrations of 11 elemental nutrients across a broad geographical gradient, provides a rare perspective on intraspecific variation in seed nutrient concentrations. To our knowledge, no comparable dataset exists that captures intraspecific seed nutrient variation at this spatial and ecological scale. Such breadth allows us to address patterns that remain largely unexplored, because previous studies have rarely examined seed nutrient composition across extensive geographical ranges within a wild species (but for smaller-scale studies, see De Frenne *et al.*, 2011; Wu *et al.*, 2024; Wang *et al.*, 2025).

Generally, seed nutrients were positively intercorrelated. The strongest correlation was between seed P and Mg (*r* = 0.65; Fig. 2), probably because their uptake and transport are partly coordinated (Weih et al., 2021) and because most seed P is stored as phytic acid, which binds cations, including Mg (Wu *et al.*, 2009). Generally, nutrient loading into seeds is often co-regulated during seed development (Himelblau and Amasino, 2001).

We found negative correlations between some seed nutrients, particularly Ca, K and Ni, and the growth-related traits, particularly seed weight. In other words, larger seeds had lower concentrations of these elements. Similar trade-offs between seed weight and protein content have been reported in cereals (Uauy *et al.*, 2006) and in the model species *Arabidopsis thaliana* (Chardon *et al.*, 2014). This relationship has been linked to the accumulation of carbohydrates in the endosperm; specifically, larger seeds have more carbohydrates and thus lower concentrations of elemental nutrients (Sehgal *et al.*, 2018).

The concentration of most elemental nutrients in seeds increased with mean annual temperature. This relationship was driven, in part, by decreasing seed weight towards warmer regions, particularly for seed elemental nutrients that were negatively correlated with seed weight (Ca, K and Ni; Supplementary Data Fig. S5). Plants from warmer areas had higher concentrations of these nutrients, probably because they had lower relative concentrations of carbohydrates. However, concentrations of nutrients that were unrelated to seed weight also increased with mean annual temperature (Mg, P, S, Mn, Ni and Zn). The positive effect of mean annual temperature on nutrient concentrations aligns with findings of a recent study on two grass species along a latitudinal gradient in China (Wang *et al.*, 2025) and with a study on a European forest herb (De Frenne *et al.*, 2011).

Soil characteristics had only a limited effect on nutrient concentrations in the seeds. The strongest effect was of soil pH, which negatively affected the concentration of Fe and Mn and positively affected Mo. The negative effects of pH on Fe and Mn are not surprising, because the bioavailability of Fe and Mn is higher in acidic environments (Sims, 1986; Ramzani *et al.*, 2016). Likewise, Mo is more available in alkaline soils (Rana *et al.*, 2025). Surprisingly, soil available nutrients, specifically nitrate, phosphate and sulphate, did not affect the concentration of the respective elements in the seeds and had, in general, only a very small effect on seed concentration of other elemental nutrients. This contrasts with ample literature on crops showing that soil nutrient availability is crucial for nutrient concentrations in the seeds (Marschner, 2012; Joy *et al.*, 2015; Wortmann *et al.*, 2018). As mentioned above, one possible explanation is the high mobility of nitrate and sulphate in the soil (Bünemann and Condron, 2007; Cameron *et al.*, 2013). Another reason might be the pooling of soil per population, which prevented us from capturing fine-scale mosaics in soil characteristics experienced by individual plants. However, our results are in line with the few existing studies on wild species showing that seed nutrient concentration across large geographical gradients is highly variable and only weakly correlated with soil properties (Wu *et al.*, 2024; Wang *et al.*, 2025).

### Population differentiation

Although many environmental predictors were significantly associated with growth-related traits and concentrations of elemental nutrients in the seeds, they explained only 4.9–12.4 and 3.3–22 % of the variation, respectively. Much more variation (32–34 and 20–45 %, respectively) was explained by population identity, independent of environmental factors. In other words, plants growing in one population had more similar traits than predicted by environmental conditions.

One possible explanation is that our environmental variables did not fully capture site-specific conditions experienced by individual plants. However, the most important environmental drivers of plant traits across large geographical scales are associated with climate (Lemke *et al.*, 2015) and are thus unlikely to underlie the large proportion of variability explained solely by the site identity. It is more likely that the similarity of plants growing at one site is caused by genetic factors. *Hordeum murinum* is predominantly self-pollinating, hence most plants at one site are likely to be close relatives, with identical or nearly identical genetic backgrounds (Volis *et al.*, 2011). It is also possible that the variability associated with environmental predictors, particularly climate, might be genetically underpinned, at least in part, because climatic gradients might covary with genetic differentiation (Leiblein-Wild and Tackenberg, 2014). However, given that the present data were measured *in situ*, it is impossible to disentangle the contributions of genetic variation and direct response to the environment (plasticity) to the variation in plant traits. This will require further research, including growing plants in a common environment, optimally in combination with molecular genetic analysis.

## Supplementary Material

mcag164_Supplementary_Data

## Data Availability

Raw data on plant traits, elemental nutrients analysed in the seeds, local environmental conditions and climate data were deposited in Zenodo (https://zenodo.org/records/20743992).

## SUPPLEMENTARY DATA

Supplementary data are available at *Annals of Botany* online and consist of the following. Figure S1: ploidy level of tested individuals of sampled *Hordeum murinum* populations across the European range. Figure S2: differences between diploids and tetraploids in seed weight. Figure S3: principal component analysis of ploidy level distribution linked with climatic and environmental conditions. Figure S4: Pearson’s correlation matrix between all downloaded CHELSA climate variables available used to select mean annual temperature, temperature seasonality and annual precipitation as non-collinear predictors. Figure S5: intraspecific variation in concentrations of elemental nutrients in the seed, results of linear mixed model testing for the effect of seed weight, climate and environment. Table S1: differences between the two ploidy levels in plant morphological traits and seed nutrient content. Table S2: population differentiation in growth-related traits and seed nutrient contents. Table S3: intraspecific variation in plant phenology and growth-related traits, results of the spatial lag model and linear mixed model, respectively, testing for the effect of climate and environment. Table S4: intraspecific variation in seed macronutrient content, results of linear mixed model testing for the effect of climate and environment. Table S5: intraspecific variation in seed micronutrient content, results of linear mixed model testing for the effect of climate and environment.

## APPENDIX

### Ploidy levels in *Hordeum murinum*: methods

For the ploidy test, we included the majority of populations, except 16 populations from the northern part of the distribution that were <10 km apart from another population, because we expected and later confirmed that there are only tetraploids in this region. Given the large number of samples to be tested (in total, >2300 plants), we implemented a stratified approach. In the first step, we screened all populations to detect regions where both ploidies occur. To do this, we analysed one random plant per population. We detected the presence of diploids only in southern Europe. Thus, for all populations south of 47°, we then analysed six to ten random individual plants pooled into one sample. If this procedure indicated the presence of both ploidy levels within one population, we verified this by analysing each of the six to ten plants individually.

We measured the ploidy using a flow cytometer (CyFlow™ Ploidy Analyser, Sysmex Europe SE). To do this, we sowed the seeds from sampled mother plants in quick pots, grew seedlings, and collected ∼1 cm leaf samples. We placed the leaf samples in a 5.5-cm-diameter Petri dish and chopped them with a razor into small pieces with two drops of CyStain™ UV Ploidy staining solution (Sysmex Europe SE, Norderstedt, Germany). After chopping, we added 2 mL of staining solution to the leaf mass and transferred the mixture to 3.5 mL sample tubes fitted with a CellTrics 50 µm filter mesh (Sysmex Europe SE, Norderstedt, Germany) to remove plant debris, leaving only the filtered cell suspension. Before we loaded the sample into the flow cytometer, we loaded reference samples (diploid, *H. murinum glaucum* accession BCC2017, which was regenerated from NGB90348, and tetraploid, *H. murinum murinum* accession BCC2009) to determine the peak position of both ploidy levels. With the pre-installed Windows™-based FCM software CyView v.1.8 (Sysmex Europe SE, Norderstedt, Germany), we were able to perform real-time data display and analysis. Each sample was classified as either diploid or tetraploid based on the similarity of the projected peaks to the reference samples with confirmed ploidy levels.

### Geographical distribution of ploidy levels

We found that diploid plants (*H. murinum* subsp. *glaucum*) were present exclusively in Spain (mainland), Morocco and Algeria in our collection (Supplementary Data Fig. S1). From the 47 populations sampled in this region, 16 were only tetraploids, 11 were only diploids, and 20 populations had mixed ploidy (Supplementary Data Fig. S2). For the main study, we retained only the 16 fully tetraploid populations from this region.

### Trait differences between ploidy levels

To test for *in situ* differences between diploids and tetraploids, we focused only on populations from the region where both ploidies grew next to each other (red square in Supplementary Data Fig. S1). We related growth-related traits (plant height, number of spikes and seed weight) and seed nutrient contents (macro- and micronutrients) as response variables to ploidy level as the fixed effect and population identity as the random effect factor, in a linear mixed model using the *lme4* package (Bates *et al.*, 2015). We found that tetraploids had heavier seeds. We found no differences between ploidy levels in any other trait (Supplementary Data Table S1).

### Habitat preferences of ploidy levels

To illustrate niche differences between the two ploidy levels [diploid (2*x*) and tetraploid (4*x*)], we performed a principal component analysis with the climatic and environmental factors (Supplementary Data Fig. S3) and coloured the individual plants according to their ploidy level.

The first two principal component axes, PC1 and PC2, explained 30.61 and 18.95 % of the total variance, respectively. PC1 showed a strong positive association with annual precipitation and negative association with temperature seasonality, soil sulphate and soil pH. PC2 was associated with soil nitrate and mean annual temperature. We found partial clustering of diploids and tetraploids, particularly along the PC1 axis. Tetraploids were partly associated with higher annual precipitation, lower pH and, potentially, lower temperature seasonality. In contrast, diploids were predominantly associated with higher temperature seasonality, higher soil sulphate, phosphate and pH levels, but lower annual precipitation (Supplementary Data Fig. S3).

This visualization indicates that diploids tend to occur in slightly drier environments and higher temperature seasonality, whereas tetraploids are more frequent in regions with higher precipitation.
